# Refractive Error, Visual Acuity and Causes of Vision Loss in Children in Shandong, China. The Shandong Children Eye Study

**DOI:** 10.1371/journal.pone.0082763

**Published:** 2013-12-23

**Authors:** Jian Feng Wu, Hong Sheng Bi, Shu Mei Wang, Yuan Yuan Hu, Hui Wu, Wei Sun, Tai Liang Lu, Xing Rong Wang, Jost B. Jonas

**Affiliations:** 1 Department of Ophthalmology, Shandong University of Traditional Chinese Medicine, Jinan, Shandong, China; 2 Eye Institute of Shandong University of Traditional Chinese Medicine, Jinan, Shandong, China; 3 School of Public Health, Shandong University, Jinan, Shandong, China; 4 The Second Affiliated Hospital of Shandong University of Traditional Chinese Medicine, Jinan, Shandong, China; 5 Department of Ophthalmology, Medical Faculty Mannheim of the Ruprecht-Karls-University, Heidelberg, Germany; Dalhousie University, Canada

## Abstract

**Purpose:**

To examine the prevalence of refractive errors and prevalence and causes of vision loss among preschool and school children in East China.

**Methods:**

Using a random cluster sampling in a cross-sectional school-based study design, children with an age of 4–18 years were selected from kindergartens, primary schools, and junior and senior high schools in the rural Guanxian County and the city of Weihai. All children underwent a complete ocular examination including measurement of uncorrected (UCVA) and best corrected visual acuity (BCVA) and auto-refractometry under cycloplegia. Myopia was defined as refractive error of ≤−0.5 diopters (D), high myopia as ≤−6.0D, and amblyopia as BCVA ≤20/32 without any obvious reason for vision reduction and with strabismus or refractive errors as potential reasons.

**Results:**

Out of 6364 eligible children, 6026 (94.7%) children participated. Prevalence of myopia (overall: 36.9±0.6%;95% confidence interval (CI):36.0,38.0) increased (*P*<0.001) from 1.7±1.2% (95%CI:0.0,4.0) in the 4-years olds to 84.6±3.2% (95%CI:78.0,91.0) in 17-years olds. Myopia was associated with older age (OR:1.56;95%CI:1.52,1.60;*P*<0.001), female gender (OR:1.22;95%CI:1.08,1.39;*P* = 0.002) and urban region (OR:2.88;95%CI:2.53,3.29;*P*<0.001). Prevalence of high myopia (2.0±0.2%) increased from 0.7±0.3% (95%CI:0.1,1.3) in 10-years olds to 13.9±3.0 (95%CI:7.8,19.9) in 17-years olds. It was associated with older age (OR:1.50;95%CI:1.41,1.60;P<0.001) and urban region (OR:3.11;95%CI:2.08,4.66);*P*<0.001). Astigmatism (≥0.75D) (36.3±0.6%;95%CI:35.0,38.0) was associated with older age (*P<*0.001;OR:1.06;95%CI:1.04,1.09), more myopic refractive error (*P<*0.001;OR:0.94;95%CI:0.91,0.97) and urban region (*P<*0.001;OR:1.47;95%CI:1.31,1.64). BCVA was ≤20/40 in the better eye in 19 (0.32%) children. UCVA ≤20/40 in at least one eye was found in 2046 (34.05%) children, with undercorrected refractive error as cause in 1975 (32.9%) children. Amblyopia (BCVA ≤20/32) was detected in 44 (0.7%) children (11 children with bilateral amblyopia).

**Conclusions:**

In coastal East China, about 14% of the 17-years olds were highly myopic, and 80% were myopic. Prevalence of myopia increased with older age, female gender and urban region. About 0.7% of pre-school children and school children were amblyopic.

## Introduction

The prevalence of myopia has profoundly increased in East and Southeast Asia and myopia has become one of the most common causes for visual impairment in these regions [1], [2]. Since myopia can be associated with major ophthalmic diseases such as myopic retinopathy and myopic glaucomatous optic neuropathy, the increased prevalence of myopia indicates an increased risk of myopia-induced visual impairment [3], [4]. Numerous studies previously examined the prevalence of myopia. These studies were carried out either in countries others than mainland China [5]–[21], which due to its population size is one of the main target countries in the research on myopia prevalence and its prevention, or the studies were performed ten or more years ago [22]–[25] or were conducted 5 to 10 years ago in South China or West China [26], [27]. There were only three recent studies on the prevalence of myopia in children in China, one was performed without cycloplegia [28], and the two other ones were conducted in Western China or Northern China far from the industrialized coastal regions of China [29], [30]. One of the characteristics of myopia in China is that its prevalence still appears to be increasing, and in particular, that the prevalence of high myopia is increasing even more markedly. It shows the importance of having updated recent data on the actual prevalence of myopia in the country. A recent study on university students in Shanghai/China and recent investigations on military conscripts in urban Seoul/Korea as well as in rural Korean regions revealed that more than 95% of the study populations were myopic, and that about 10–20% were highly myopic (myopia refractive error >−6 diopters) [31]–[33]. Also, there has been little information about regional differences in the prevalence of myopia in China. We therefore conducted this study to examine the prevalence of myopia in a school-based investigation in the East Chinese province of Shandong in a rural region and an urban area. Since myopia is associated with visual impairment, we additionally measured visual acuity and assessed the causes for visual impairment. Methods.

## Methods

### Ethics Statement

The Ethics Board of the Eye Institute of the Shandong University of Traditional Chinese Medicine and the local Administration of the Education and School Board approved the study and informed written consent was obtained from the parents or guardians of all children.

The Shandong Children Eye Study was a cross-sectional, school-based study which was performed in the city of Weihai in the most Eastern part, and in the county of Guanxian in the most Western part of the coastal province of Shandong in East China. According to the data from the census of 2010, the total population of the city of Weihai was 2,804,800, and the total population of the rural region of Guanxian was 764,900 [34]. In an attempt to cover the whole range of socioeconomic background, we chose the rural county of Guanxian in Western Shandong and the relatively highly developed city of Weihai in Eastern Shandong as study sites, since both differed markedly in their level of social and economic development.

A stratified cluster sampling method was applied. The sampling frame was based on the enumeration of grade-specific classes within the schools and kindergardens. Stratification of clusters by grade and age ensured that all ages from 4 years to 18 years were representatively included into the study samples. The sample size was calculated by estimating an age-specific prevalence of refractive errors of 33.5% with a 20% error rate and a 95% confidence interval. Assuming a non-response rate of 10%, the cluster sample size was 210 for each age, so that 3150 children with an age from 4 years to 18 years old of the two regions should be enrolled into the survey. Since there were about 50 students per class, 127 classes were included. For each grade, the classes were drawn by a simple random sampling and all students in these classes were asked to participate in the study.

Before the examinations were carried out, an interview was performed using a standardized questionnaire to obtain information of the children's family history, time spent doing outdoor activities and indoor activities, study intensity, history of previous eye examinations and treatments, lifestyle, etc. The questionnaire was similar to the questionnaire used previously in the RESC (Refractive Error Study in Children) studies [35], [36]. The first step of the series of examinations consisted of the assessment of uncorrected visual acuity (UCVA), for which a tumbling “E” chart (#600722, Good-Lite Co., Elgin, IL, USA) was used at a distance of 3 m. The lowest line of the chart and the eyes of the tested children were approximately at the same height. The children were asked to start with the upper first line (visual acuity: 20/100) of the chart, and to continue with the next lines, if at maximum one character of the line was incorrectly described. When the children falsely described at least 2 characters in the line, visual acuity was recorded as the value of the previous line. If the children could not read the 20/100 line at a distance of 3m, the test was repeated at a distance of 1m. If at that distance no line could be read, visual acuity was tested as counting fingers, hand movement, light perception or no light perception. In a second step, auto-refractometry was performed (KR-8900, Topcon, Itabashi, Tokyo, Japan). Each eye was measured at least 3 times. The difference between the maximum and minimum value of the measurements of spherical refractive error and cylindrical refractive error had to be less than 0.5 D, otherwise the measurements had to be repeated. If UCVA was less than 20/20, best corrected visual acuity (BCVA) was measured in a third step of the examination. For that purpose, the results of auto-refractometry were used as basis. To assure the quality for the measurements of visual acuity and refractometry, the examiners were repeatedly checked for the accuracy of their results. Intraocular pressure was measured by a non-contact tonometer (CT-80A, Topcon, Co., Tokyo, Japan). An ophthalmologist examined the anterior and posterior ocular segments of all children. After ensuring that there was no risk for a medical mydriasis, cycloplegia was performed. Cycloplegia was achieved by using 1% cyclopentolate eye drops (Alcon, Ft. Worth, Texas, USA). After an initial topical anesthesia with one drop of 0.4% oxybuprocaine (Santen Co., Shiga, Japan) for each eye, three drops of 1% cyclopentolate were instilled in intervals of 5 minutes. About 30 minutes after the last drop instillation, a repeated autorefractometry was performed. If a pupil diameter of at least 6 mm was not achieved, another drop of cyclopentolate was given and the examination was repeated 10 minutes later. Less than 10% of the children needed an additional drop of cyclopentolate due to an initially insufficient pupillary mydriasis and cycloplegia. To assess the causes for visual impairment, children with a BCVA <20/20 underwent a repeated ophthalmoscopical examination in medical mydriasis.

Refractive errors were defined as suggested by the Refractive Error Study in Children (RESC) surveys [35]–[36]. Myopia and hyperopia were calculated as spherical equivalent of the refractive error, defined as the sum of the spherical refractive error plus half of the cylindrical refractive error (measured as minus values). Myopia was defined per subject as refractive error (spherical equivalent) of ≤−0.50D in one or both eyes. High myopia was defined as refractive error ≤−6.0D in one or both eyes. Mild hyperopia was defined as a refractive error of >+0.50D to ≤+2.0D, and medium to marked hyperopia was defined as a refractive error of >+2.0 D, in one or both eyes if neither eye was myopic. Emmetropia was consequently considered to be a refractive error of >−0.50D and ≤+0.50D in both eyes. Astigmatism was a cylindrical refractive error ≥0.75D in either eye. Anisometropia was defined as difference between right eye to left eye in refractive error (spherical error) of ≥1.0D. All refractive errors were measured under cycloplegia.

The reasons for a reduced UCVA were assessed by refractometry and by the ophthalmological examination. Amblyopia was present if BCVA could not be improved to more than 20/32, if no other reason such as cataract could be detected as cause for the reduction in BCVA, and if factors such as strabismus, hyperopia, myopia or anisometropia could explain amblyopia. If these conditions did not prevail, a reduced BCVA was considered to be unexplained. The same definition of amblyopia was applied by in the study by Negrel and colleagues [38].

Statistical analysis was performed using a commercially available statistical software package (SPSS for Windows, version 21.0, IBM-SPSS, Chicago, IL). In a first step, we examined the mean values (presented as mean ± standard deviation). Frequencies were presented as mean ± standard error. In a second step, we performed a univariate binary regression analysis with the presence of myopia (or of hyperopia) as dependent parameter and one of the ocular parameters or one of the general parameters as independent parameter. In a third step, we carried out a multivariate binary regression analysis, with the presence of myopia (or of hyperopia) as the dependent variable and all those parameters as independent parameters which were significantly associated with the dependent variable in the univariate analysis. For continuous variables such as refractive error, we first performed a univariate analysis to search for associations. We then carried out a multivariate linear regression analysis including those variables which were significantly associated with the continuous variable in the univariate analysis. Odds ratios (OR) and 95% confidence intervals (95%CI) were calculated. All *P*-values were 2-sided and were considered statistically significant when the values were less than 0.05.

## Results

Out of the 6364 children who were primarily eligible for the study, 328 refused the examination and 10 children who had an intraocular pressure of more than 25 mmHg in one or both eyes were excluded from the survey to avoid any risk associated with cycloplegia. The study eventually included 6026 (94.7%) children (3186 (52.9%) boys) with a mean age of 9.7±3.3 years (range 4 to 18 years) (Table 1). Out of the 6026 children, 3112 children lived in the rural Guanxian County (51.6%) and 2914 children came from the city of Weihai (48.4%). Except for a boy with phthisis bulbi in his left eye, all children underwent bilateral cycloplegic refractive error measurements.

**Table 1:** Enumerated and Examined Population Stratified by Age in the Shandong Children Eye Study.

Age (Years)	Number (%) of Enumerated Population	Number (%) of Examined Population	Percentage of Examined (%)
4	129 (2.0)	115 (1.9)	89.1
5	388 (6.1)	361 (6.0)	93.0
6	465 (7.3)	444 (7.4)	95.5
7	674 (10.6)	642 (10.7)	95.3
8	770 (12.1)	745 (12.4)	96.8
9	567 (8.9)	550 (9.1)	97.0
10	728 (11.4)	705 (11.7)	96.8
11	610 (9.6)	590 (9.8)	96.7
12	496 (7.8)	488 (8.1)	98.4
13	446 (7.0)	439 (7.3)	98.4
14	359 (5.6)	342 (5.7)	95.3
15	267 (4.2)	232 (3.9)	86.9
16	155 (2.4)	136 (2.3)	87.7
17	158 (2.5)	130 (2.2)	82.3
18	152 (2.4)	107 (1.8)	70.4
**Total**	6364 (100.0)	6026 (100.0)	94.7

The mean spherical equivalent of the right eyes was −0.22±2.06 D (median: 0.38 D; range: −11.75D to +10.5D) and of the left eyes −0.13±2.05 D (median: 0.50 D; range: −11.75D to +11.25D). The mean spherical equivalent of the worse eye was −0.18±2.11 D (median: 0.50 D; range: −11.75D to +11.25D).

**Figure 1:**
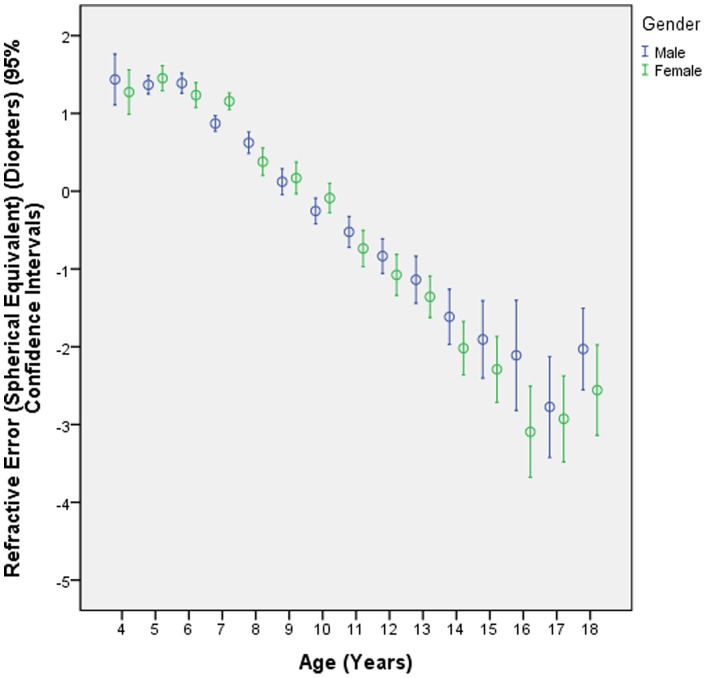
Distribution of the refractive error (spherical equivalent) of right eyes, stratified by age and gender in the Shandong Children Eye Study.

**Figure 2:**
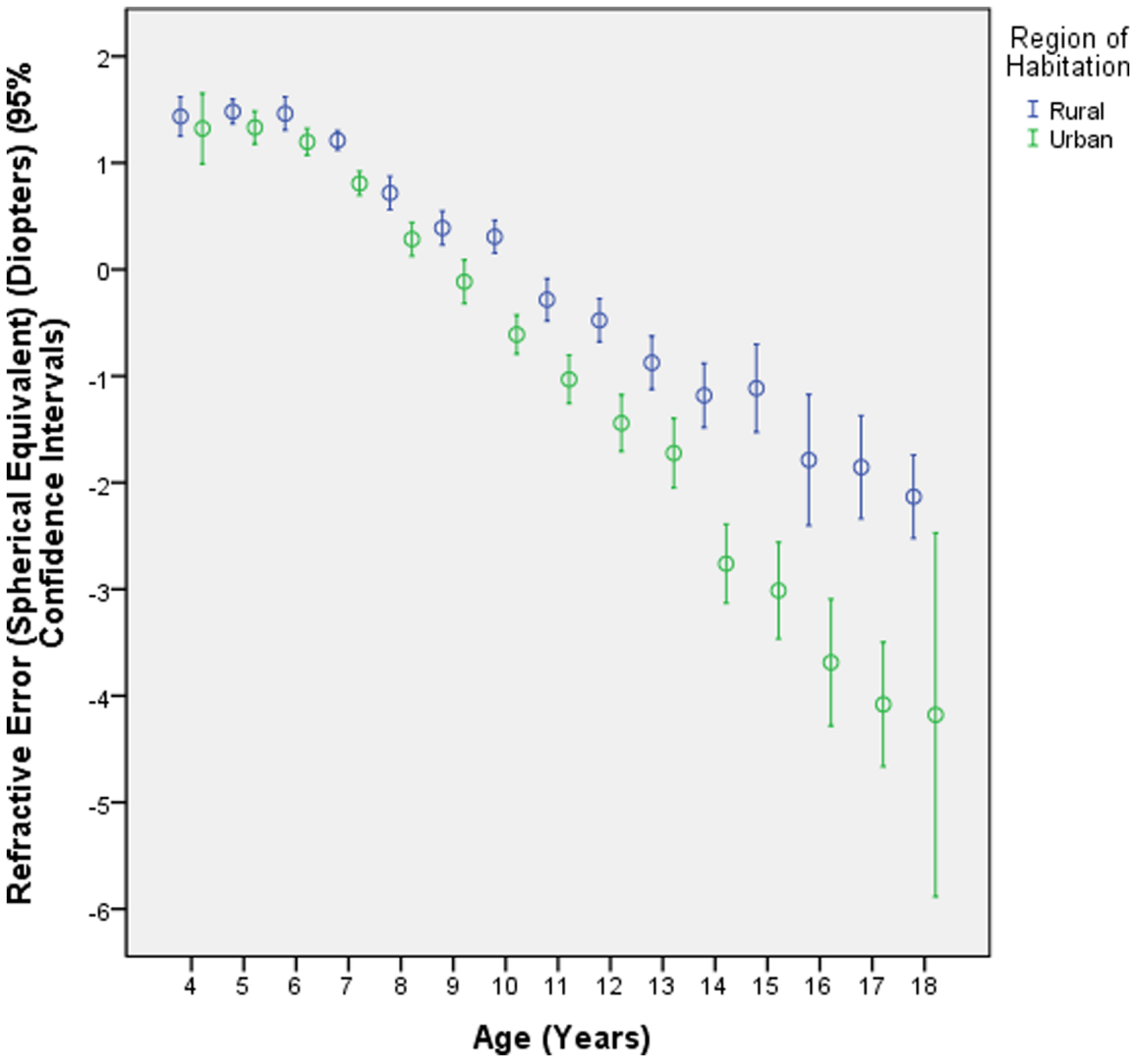
Distribution of the refractive error (spherical equivalent) of right eyes, stratified by age and region of habitation, in the Shandong Children Eye Study.

**Figure 3:**
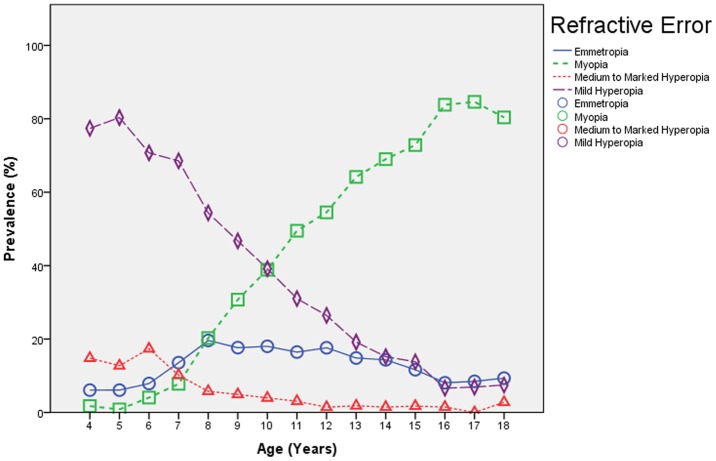
Prevalence of Medium to Marked Hyperopia (>+2.0 Diopter (D)), Mild Hyperopia (>+0.50D to ≤+2.0D), Emmetropia, Mild Myopia (≤−0.50D) and High Myopia (≤−6.0D) Stratified by Age in the Shandong Children Eye Study.

Prevalence of myopia was overall 36.9±0.6% (95%CI: 36.0, 38.0). The prevalence of myopia increased from 1.7±1.2% (95%CI: 0.0, 4.0) in the 4 years old children to 84.6±3.2% (95%CI: 78.0, 91.0) in 17-year olds (Fig. 1, 2, 3). In univariate analysis, prevalence of myopia was significantly associated with female gender (*P*<0.001) and urban region of habitation (*P*<0.001) (Fig. 1, 2). We then performed a multivariate analysis (binary regression analysis) with presence of myopia as dependent variable and age, gender and region of habitation as independent parameters. It revealed that the presence of myopia was significantly associated with older age (OR: 1.56 (95%CI: 1.52, 1.60); *P*<0.001), female gender (OR: 1.22 (95%CI: 1.08, 1.39); *P* = 0.002) and region of habitation OR: 2.88 (95%CI: 2.53, 3.29); *P*<0.001).

**Figure 4:**
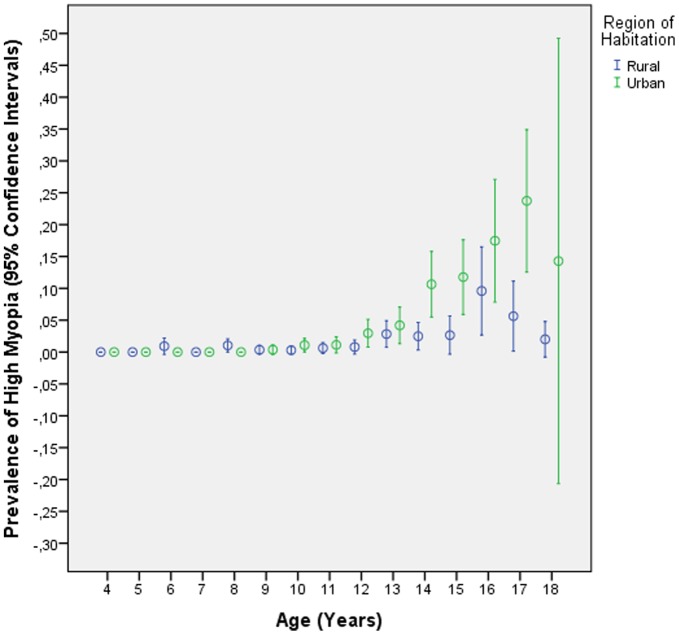
Prevalence of High Myopia (Defined as Refractive Error ≤−6.0 Diopters) Stratified by Age and Region of Habitation in the Shandong Children Eye Study.

The prevalence of high myopia was 2.0±0.2%. It increased significantly from 0% in the 4-years old, to 0.7±0.3% (95%CI: 0.1, 1.3) in the 10-years olds, to 5.9±1.3% (95%CI: 3.4, 8.4) in the 14-years olds, and to 13.9±3.0 (95%CI: 7.8, 19.9) in the 17-years olds (Tables 2, 3, 4) (Fig. 4). Prevalence of high myopia was additionally associated with female gender (*P*<0.001) and urban region of habitation (*P*<0.001). In multivariate analysis, prevalence of high myopia remained significantly associated with older age (OR: 1.50 (95%CI: 1.41, 1.60); *P*<0.001) and region of habitation (OR: 3.11 (95%CI: 2.08, 4.66); *P*<0.001), while gender was no longer significantly (*P* = 0.25) associated.

**Table 2:** Prevalence (%) (95% confidence interval) of high myopia, myopia, hyperopia, astigmatism, and anisometropia as measured under cycloplegia among children with an age of 4 to 18 years, stratified by age, gender and region of habitation (n: Number of children).

Age (years)	Total Number	High Myopia		Myopia		Mild Hyperopia >+0.5D, ≤+2.0D		Medium to Marked Hyperopia >+2.0D		Astigmatism		Anisometropia	
		n	Prevalence (%)	n	Prevalence (%)	n	Prevalence (%)	n	Prevalence (%)	n	Prevalence (%)	n	Prevalence (%)
4	115	0	−	2	1.7 (0.3–5.3)	89	77.4(69.2–84.4)	17	14.8(9.1–22.0)	40	34.8 (26.5–43.8)	4	3.5 (1.1–7.9)
5	361	0	−	3	0.8 (0.2–2.1)	290	80.3(76.0–84.2)	46	12.7(9.6–16.4)	107	29.6 (25.1–34.5)	7	1.9 (0.8–3.7)
6	444	2	0.5 (0.1–1.4)	18	4.1 (2.5–6.2)	314	70.7(66.4–74.8)	77	17.3(14.0–21.0)	139	31.3 (27.1–35.7)	11	2.5 (1.3–4.2)
7	642	0	−	50	7.8 (5.9–10.0)	440	68.5(64.9–72.0)	65	10.1(8.0–12.6)	206	32.1 (28.6–35.8)	18	2.8 (1.7–4.3)
8	745	4	0.5 (0.2–1.2)	151	20.3 (17.5–23.3)	405	54.4(50.8–57.9)	43	5.8(4.2–7.6)	237	31.8 (28.5–35.2)	25	3.4 (2.2–4.8)
9	550	2	0.4 (0.1–1.1)	169	30.7 (27.0–34.7)	257	46.7(42.6–50.9)	27	4.9(3.3–6.9)	187	34.0 (30.1–38.0)	33	6.0 (4.2–8.2)
10	705	5	0.7 (0.3–1.5)	274	38.9 (35.3–42.5)	276	39.1(35.6–42.8)	28	4.0(2.7–5.6)	232	32.9 (29.5–36.4)	46	6.5 (4.9–8.5)
11	590	5	0.8 (0.3–1.8)	292	49.5 (45.5–53.5)	183	31.0(27.4–34.8)	18	3.1(1.9–4.6)	198	33.6 (29.8–37.4)	41	6.9 (5.1–9.2)
12	488	9	1.8 (0.9–3.3)	266	54.5 (50.1–58.9)	129	26.4(22.7–30.5)	7	1.4(0.6–2.8)	186	38.1 (33.9–42.5)	38	7.8 (5.6–10.4)
13	438	15	3.4 (2.0–5.4)	281	64.2 (59.6–68.6)	84	19.2(15.7–23.0)	8	1.8(0.8–3.4)	179	40.9 (36.3–45.5)	49	11.2 (8.2–14.4)
14	342	20	5.8 (3.7–8.7)	236	69.0 (64.0–73.8)	52	15.2(11.7–19.3)	5	1.5(0.5–3.1)	155	45.3 (40.1–50.6)	51	14.9 (11.4–18.9)
15	232	17	7.3 (4.4–11.2)	169	72.8 (66.9–78.3)	32	13.8(9.8–18.6)	4	1.7(0.5–4.0)	124	53.4 (47.0–59.8)	34	14.7 (10.5–19.6)
16	136	18	13.2 (8.2–19.6)	114	83.8 (77.0–89.4)	9	6.6(3.2–11.6)	2	1.5(0.2–4.5)	73	53.7 (45.3–61.9)	21	15.4 (10.0–22.1)
17	130	18	13.8 (8.6–20.5)	110	84.6 (77.8–90.1)	9	6.9(3.4–12.1)	0	−	64	49.2 (40.7–57.8)	24	18.5 (12.4–25.7)
18	107	3	2.8 (0.7–7.1)	86	80.4 (72.2–87.1)	8	7.5(3.5–13.5)	3	2.8(0.7–7.1)	60	56.1(46.6–65.3)	18	16.8 (10.6–24.7)

**Table 3:** Prevalence (95% confidence intervals) of high myopia, myopia, hyperopia, astigmatism, and anisometropia as measured under cycloplegia among children with an age of 4 to 18 years, stratified by age, gender and region of habitation (n: Number of children).

Gender	Number	High Myopia		Myopia		Mild Hyperopia >+0.5D, ≤+2.0D		Medium to Marked°Hyperopia >+2.0D		Astigmatism		Anisometropia	
		n	Prevalence (%)	n	Prevalence (%)	n	Prevalence (%)	n	Prevalence (%)	n	Prevalence (%)	n	Prevalence (%)
Male	3186	51	1.6 (1.2–2.0)	1079	33.9 (32.2–35.5)	1432	44.9(43.2–46.7)	184	5.8(5.0–6.6)	1129	35.4 (33.8–37.1)	206	6.5 (5.6–7.4)
Female	2839	67	2.4 (1.8–3.0)	1142	40.2 (38.4–42.0)	1145	40.3(38.5–42.1)	166	5.8(5.0–6.8)	1058	37.3 (35.5–39.1)	214	7.5 (6.6–8.5)
**Region**													
Rural	3111	40	1.3 (0.9–1.7)	954	30.7 (29.1–32.3)	1531	49.2(47.5–51.0)	199	6.4 (5.6–7.3)	1000	32.1 (30.5–33.8)	189	6.1 (5.3–7.0)
Urban	2914	78	2.7 (2.1–3.3)	1267	43.5 (41.7–45.3)	1046	35.9(34.2–37.6)	151	5.2 (4.4–6.0)	1187	40.7 (39.0–42.5)	231	7.9 (7.0–8.9)
**Total**	6025	118	2.0 (1.6–2.3)	2221	36.9(35.6–38.1)	2577	42.8(41.5–44.0)	350	5.8 (5.2–6.4)	2187	36.3 (35.1–37.5)	420	7.0 (6.4–7.7)

**Table 4:** Mean refractive error (mean ± standard deviation) in the Shandong Children Eye Study stratified by age, gender and region of habitation (n: Number of children).

Age (Years)	Gender	Region of Habitation	n	Refractive Error (Diopters) Mean ± Standard Deviation
4	Boys	Rural	25	1.37±0.47
		Urban	38	1.50±1.64
	Girls	Rural	17	1.57±0.73
		Urban	35	1.13±1.13
5	Boys	Rural	105	1.43±0.82
		Urban	103	1.30±0.93
	Girls	Rural	69	1.55±0.68
		Urban	84	1.37±1.22
6	Boys	Rural	136	1.56±1.08
		Urban	128	1.21±1.02
	Girls	Rural	81	1.29±1.29
		Urban	98	1.19±0.88
7	Boys	Rural	181	1.17±0.79
		Urban	182	0.57±1.07
	Girls	Rural	118	1.27±0.79
		Urban	161	1.07±0.97
8	Boys	Rural	207	0.86±1.28
		Urban	178	0.35±1.46
	Girls	Rural	174	0.54±1.81
		Urban	186	0.22±1.59
9	Boys	Rural	172	0.44±1.31
		Urban	120	−0.34±1.50
	Girls	Rural	111	0.30±1.41
		Urban	147	0.07±1.85
10	Boys	Rural	181	0.19±1.36
		Urban	178	−0.71±1.68
	Girls	Rural	155	0.44±1.52
		Urban	190	−0.52±1.88
11	Boys	Rural	164	−0.23±1.70
		Urban	145	−0.85±1.81
	Girls	Rural	157	−0.34±1.93
		Urban	124	1.24±1.96
12	Boys	Rural	130	−0.47±−1.57
		Urban	133	−1.19±2.01
	Girls	Rural	121	−0.49±1.70
		Urban	104	−1.76±2.13
13	Boys	Rural	120	−0.73±2.15
		Urban	107	−1.60±2.43
	Girls	Rural	128	−10.01±1.84
		Urban	84	−1.88±2.07
14	Boys	Rural	94	−0.94±2.07
		Urban	65	−2.59±2.22
	Girls	Rural	107	1.40±2.24
		Urban	75	−2.91±2.22
15	Boys	Rural	58	−0.64±2.15
		Urban	64	−3.05±2.81
	Girls	Rural	55	−1.61±2.19
		Urban	55	−2.97±2.10
16	Boys	Rural	36	−1.29±2.60
		Urban	23	−3.39±2.43
	Girls	Rural	37	−2.27±2.61
		Urban	40	−3.86±2.33
17	Boys	Rural	37	−2.02±1.97
		Urban	15	−4.63±2.14
	Girls	Rural	34	−1.68±2.13
		Urban	44	−3.89±2.27
18	Boys	Rural	55	−1.90±1.94
		Urban	4	−3.81±2.39
	Girls	Rural	45	−2.42±1.98
		Urban	3	−4.67±1.01

The prevalence of mild hyperopia was 42.8% (95%CI: 41.5, 44.0). In univariate analysis, it decreased significantly with older age (*P*<0.001), urban region of habitation (*P*<0.001) and female gender (*P*<0.001). In binary regression analysis, prevalence of mild hyperopia remained significantly associated with younger age (OR: 0.72 (95%CI: 0.70, 0.73); *P*<0.001), rural region of habitation (OR: 0.58 (95%CI: 0.52, 0.64); *P*<0.001) and male gender (OR: 0.83 (95%CI: 0.75, 0.92); *P*<0.001).

The prevalence of medium to marked hyperopia was 5.8% (95%CI: 5.2, 6.4). In univariate analysis, it decreased significantly with older age (*P*<0.001) and urban region of habitation (*P* = 0.04). It did not differ significantly (*P* = 0.91) between boys and girls. In binary regression analysis, prevalence of medium to marked hyperopia remained significantly associated with younger age (OR: 0.76 (95%CI: 0.72, 0.79); *P*<0.001) and rural region of habitation (OR: 0.80 (95%CI: 0.64, 0.99); *P* = 0.04).

Astigmatism was found in 36.3±0.6% (95%CI: 35.0, 38.0). Its mean value was 0.43±0.51 diopters (median: 0.25 diopters; range: 0.00 to 6.50 diopters). In multivariate analysis, the amount of astigmatism was associated with older age (*P*<0.001; standardized coefficient beta: 0.07; regression coefficient B: 0.01; 95%CI: 0.01, 0.02) and more myopic refractive error (*P* = 0.008; beta: −0.04; B: −0.01; 95%CI: −0.02, −0.01), while it was not associated with urban region of habitation (*P* = 0.14) nor gender (*P* = 0.08). In binary regression analysis, the prevalence of astigmatism remained significantly associated with older age (*P<*0.001; regression coefficient B: 0.06; OR: 1.06 (95%CI: 1.04, 1.09), more myopic refractive error (*P<*0.001; B: −0.07; OR: 0.94 (95%CI: 0.91, 0.97), and urban region of habitation (*P<*0.001; B: 0.38; OR: 1.47 (95%CI: 1.31, 1.64).

Prevalence of anisometropia was 7.0±0.3% (95%CI: 6.0, 8.0). It was associated with older age (*P*<0.001), myopic refractive error (*P*<0.001), and urban region of habitation (*P* = 0.005) while it was not associated with gender (*P* = 0.10). In binary regression analysis, prevalence of anisometropia remained significantly associated with older age (OR: 1.22 (95%CI: 1.19, 1.26); *P*<0.001) and urban region (OR: 1.55 (95%CI: 1.26, 1.90); *P*<0.001), while it was no longer significantly associated with refractive error (*P* = 0.37).

Out of the 6026 children who underwent cycloplegic refractometry, 18 children (age: 4 and 5 years) did not sufficiently cooperate for a reliable visual acuity test, so that data of visual acuity measurements were eventually available for 6008 children (Table 5). Out of these 6008 children, 1628 (27.1%) children had an uncorrected visual acuity (UCVA) of 20/40 or worse in the better eye, and 156 (2.6%) children had an UCVA of 20/200 or less in the better eye (Table 5). UCVA was significantly associated with female gender (OR: 1.36 (95%CI: 1.22–1.53; *P*<0.001) and rural region (*P*<0.001; OR: 1.75 (95%CI: 1.56–1.96). Using the same categorization as the one used by Xiang and colleagues, a normal vision (UCVA in the better eye ≥6/6) was achieved by 60.2% children, a mildly reduced visual acuity (6/9<UCVA<6/6) by 7.8% of the children, a moderately reduced visual acuity (6/18<UCVA ≤6/9) by 12.2% of the children, and a severely reduced visual acuity (UCVA ≤6/18) by 19.7% of the children [39]. UCVA strongly decreased with older age (*P*<0.001; regression coefficient B: −0.05; standardized regression coefficient r: −0.48).

**Table 5:** Distribution of uncorrected and best corrected visual acuity in each visual acuity category, given in number, percentage and 95% confidence interval (CI).

Visual Acuity Category	Uncorrected Visual Acuity		Best Corrected Visual Acuity	
	Number	Percentage (95%CI)	Number	Percentage (95%CI)
≥20/32 in both eyes	3962	65.8 (64.6–67.0)	5938	98.8(98.5–99.1)
≥20/32 in one eye	418	7.0 (6.3–7.6)	51	0.8 (0.6–1.1)
20/40 to 20/63 in better eye	684	11.4 (10.6– 12.2)	17	0.3 (0.2–0.4)
20/80 to 20/160 in better eye	788	13.1 (12.2–13.9)	2	0.03(0.01–0.1)
≤20/200 in better eye	156	2.6 (2.2–3.0)	−	−
**Total**	6008	100	6008	100

Out of the 6008 children with visual acuity measurements, 2046 (34.05%) children had an UCVA of ≤20/40 in at least one eye (Table 6). Out of these 2046 children, 1975 (96.6%) children achieved a visual acuity of ≥20/32 by providing adequate correction of refractive error. Using BCVA, 19 (0.32%) children had a BCVA of ≤20/40 in the better eye, and there was no child with a BCVA ≤20/200 in the better eye. BCVA was significantly higher in the urban region of habitation (*P*<0.001) and did not differ significantly (*P* = 0.49) between boys and girls. Amblyopia (defined as BCVA ≤20/32, explained by factors such as strabismus or refractive errors, and no morphological reason for BCVA reduction) was the reason for reduced visual acuity in 44 children (2.15% of the 2046 or 0.73% of the total study population), with 11 children having bilateral amblyopia. Nine of these children had bilateral hyperopia and two children were highly myopic in both eyes with a myopic refractive error of ≤−7.38 D and ≤−11.00 D in both eyes, respectively. In 33 children, amblyopia was unilateral with hyperopia being the cause in 10 children and anisometropia in 23 children. Other causes of reduced vision were congenital cataract, corneal opacity due to keratitis, lens injury, bilateral optic nerve atrophy and unilateral phthisis bulbi. Visual impairment in 28 eyes of 22 children remained unexplained.

**Table 6:** Causes of Uncorrected Visual Acuity (≤20/40) in the Shandong Children Eye Study.

Cause of Uncorrected Visual Acuity of ≤20/40	Right Eyes*	Left Eyes*	Number of Children*	Percentage of Children with Uncorrected Visual Acuity ≤20/40 in the Total Study Population
Undercorrected Refractive Error	1810 (97.6%)	1774 (97.5%)	1975 (96.6%)	32.87%
Amblyopia	29 (1.56%)	26 (1.43%)	44 (2.15%)	0.73%
Corneal Opacity	0 (0%)	1 (0.05%)	1 (0.05%)	0.02%
Lens Disease	1 (0.05%)	2 (0.11%)	2 (0.10%)	0.03%
Fundus Disorder	1 (0.05%)	1 (0.05%)	1 (0.05%)	0.02%
Other Disease	0 (0%)	1 (0.05%)	1 (0.05%)	0.02%
Unexplained	14 (0.75%)	14 (0.77%)	22 (1.08%)	0.37%
**Total**	1855 (100%)	1819 (100%)	2046 (100%)	34.05%

* : % refers to the total group of children with uncorrected visual acuity of ≤20/40 (n = 2046 children).

## Discussion

Our school-based study was performed in a rural region and in a city in the East Chinese province of Shandong. The overall prevalence of myopia was 36.9±0.6% and increased from 1.7±1.2% in the 4 years olds to 84.6±3.2% in 17-year olds. Prevalence of myopia was associated with female gender, urban region of habitation and increasing age. The prevalence of high myopia was 2.0±0.2% and increased from 0.7±0.3% in 10-years olds to 13.9±3.0 in the 17-years olds. As myopia in general, high myopia was associated with urban region but not with gender. Astigmatism was found in 36.3±0.6% of the children, and it was associated with older age, urban region and more myopic refractive error. Anisometropia was detected in 7.0±0.3% of the study population in association with older age and urban region.

These results agree with previous population-based and school-based investigations in China documenting a marked increased prevalence of myopia in the younger generation [22]–[27]. It is in contrast to other countries such as Laos, Iran, South Africa, Morocco, Brazil and Poland and Scandinavian countries, in which either no increased prevalence of myopia or a considerably less marked increase in the prevalence of myopia has been reported [5], [8], [11]–[18], [21], [40], [41]. The prevalence of low to medium myopia was markedly higher in the present Shandong Children Eye Study than in the previous study on adults in the neighboring region of Greater Beijing giving another example for the increased prevalence of myopia across the generations [42]. Correspondingly, a recent study from Beijing showed that the refractive error of children at the age of 11 years was similar to that of their parents, while children at the age of 18 years were up to 2.0 D more myopic than their parents [43].

The prevalence of high myopia of 13.9% in the 17-year-old children of our study was higher than in most previous children studies, and it was markedly higher than in the Beijing Eye Study on adults (2.6%; 95%CI: 2.2, 3.1). Interestingly, the prevalence of high myopia showed a particular pattern, with the prevalence of high myopia starting to increase around the age of 10–12 years (Fig. 3). This pattern has also been previously reported from Taiwan and from Guangzhou, and Xiang and colleagues have suggested that it should be called “acquired high myopia”, because the age of onset roughly corresponded to the time it would take for early onset myopia to progress to high myopia [23], [44], [45].

Since the prevalence of high myopia increased with older age, one can assume that that the prevalence of high myopia in the school children of our study will further increase when the children get older. It agrees with the high prevalence of high myopia of 20% found in recent studies on university students in Shanghai and on military conscripts in urban and rural Korea [31]–[33]. Since high myopia can lead to vision threatening ocular disorders, the impact of myopia on Public Health in China will therefore markedly increase. In the adult population in China, degenerative myopia is responsible for one third of the causes of visual impairment and is second only to cataract [1].

Out of 6008 children, 2046 (34.05%) children had an UCVA of ≤20/40 in at least one eye, with refractive error being the cause in 1975 (32.9%) children. This prevalence of 32.9% of refractive error as cause for UCVA was comparable to the figures found in the recent Global Burden of Disease Study, in which refractive error was the most common cause for UCVA [46]. Among the causes for a reduced BCVA, amblyopia was the most common one in our study population, although the prevalence of amblyopia was relatively low (0.73%). The prevalence of amblyopia of 0.73% in our study population was in agreement with the prevalence of 0.8% found in the Singaporean Preschoolers Study [47].

UCVA strongly decreased with older age in our study population. It confirms the recent study by Xiang and colleagues who estimated the prevalence of myopia based on reduced unaided visual acuity in Chinese school children in Guangzhou over the past 20 years [39]. They found that in 1988, over 80% of children in grade 1 (age 6 years) and about 30% in Grade 12 (age 17 years) had normal UCVA. By 2007, these figures dropped to only 60% in grade 1 and about 10% in Grade 12. In the period from 2003 to 2007, the figures remained unchanged at both the grade 1 and grade 12 levels. The authors concluded that the prevalence of reduced UCVA increased markedly in children from Guangzhou over the last 20 years, but stabilized in the past few years.

The overall prevalence of mild hyperopia and medium to marked hyperopia in our study was 42.8% and 5.8% respectively, and as a corollary to the prevalence of myopia, it decreased with older age (Fig. 3). Again as a corollary to the prevalence of myopia, the prevalence of hyperopia overall was associated with younger age and rural region of habitation. The decrease in the prevalence of hyperopia overall and the increase in the prevalence of myopia with increasing age of the children as found in our study population from Shandong was similar to changes as described by Morgan and colleagues in the Guangzhou “Refractive Error Study in Children” study [37]. Interestingly and in contrast to myopia, hyperopia overall was not significantly correlated with gender in our study.

Astigmatism was detected in 36.3% of the children in our survey. Its mean amount was 0.43±0.51 diopters. It was associated with older age and more myopic refractive error. The prevalence of astigmatism in our study population was relatively high, with higher rates reported only in studies from Singapore and Guangzhou [10], [23]. Potential limitations of our study should be mentioned. Firstly, Shandong providence in coastal East China is not representative of China as a whole, although our study included an urban part and a rural region. Shandong is economically less developed than the metropolitan regions of Beijing, Shanghai and Guangzhou, and it is better developed than the provinces of Inner Mongolia or Western China. Secondly, our investigation was a cross-sectional study which did not allow drawing conclusions on a longitudinal course and causal relationship between parameters. Any statement about an increase in the prevalence of myopia in our study was therefore meant only in a cross sectional manner when comparing younger children with older children. Thirdly, in a similar manner, the comparison in the prevalence of myopia in children from Shandong with the prevalence of myopia in adults from Beijing is not quite correct in view of the different locations. The comparison of the data from the children from Shandong with the data from the adults from Beijing however is however in agreement with the comparison from children and adults in Beijing and from children and adults in Guangzhou [23], [26], [48], [49], although also in the latter comparisons, issues about longitudinal changes remained resolved. Fourthly, although the response rate in our study to participate in the cycloplegic examination was relatively high with 94.7%, the group of 18-years old teenagers had a relatively low response rate of 70.4% (Table 1). It may have been the reason why the prevalence of high myopia was considerably lower in the 18-years old group than in the 17-years old group (13.8% (95%CI: 8.6, 20.5) versus 2.8% (0.7, 7.1), so that we mainly reported on the prevalence of high myopia in the 17-years old children. Fifthly, our study protocol was not completely consistent with the one applied by the Refractive Error Study in Children (RESC). We used a tumbling E chart, while the RESC used an ETDRS (Early Treatment of Diabetic Retinopathy Study) Eye Chart. Sixthly, while school participation is, for all practical purposes, universal in China, this is not true at the kindergarten level. There may therefore have been a sampling bias in the 4 and 5 year-olds. Seventhly, we defined astigmatism as a cylindrical refractive error ≥0.75D in either eye. Since other studies used a cut-off value of 1.00 diopters [6], [26], differences in the prevalence of astigmatism between our study and other investigations may also be due to the difference in the definition of astigmatism.

In conclusion, in coastal East China, about 14% of the 17-years olds were highly myopic, and 80% were myopic. Prevalence of myopia increased with older age, female gender and urban region. About 0.7% of pre-school children and school children were amblyopic. The marked increase in the prevalence of myopia, in particular of high myopia, in the young generation of China as compared to elderly population groups will be of importance for future public health politics and warrants measures to prevent the development of myopia.
